# Author Correction: Measuring exposure to misinformation from political elites on Twitter

**DOI:** 10.1038/s41467-022-35420-0

**Published:** 2022-12-13

**Authors:** Mohsen Mosleh, David G. Rand

**Affiliations:** 1 Management Department, University of Exeter Business School, Exeter, UK; 2 Alan Turing Institute, London, UK; 3 Sloan School of Management, Massachusetts Institute of Technology, Cambridge, MA USA; 4 Initiative on the Digital Economy, Massachusetts Institute of Technology, Cambridge, MA USA; 5 Department of Brain and Cognitive Sciences, Massachusetts Institute of Technology, Cambridge, MA USA

**Keywords:** Human behaviour, Communication

Correction to: *Nature Communications* 10.1038/s41467-022-34769-6, published online 21 November 2022

The original version of this Article contained an error in the Abstract, which incorrectly read:

Additionally, we analyze the co-follower, co-share, and co-retweet networks of 5000 Twitter users and find an ideological asymmetry: estimated ideological extremity is associated with more misinformation exposure for users estimated to be conservative but not for users estimated to be liberal.

The correct version replaces this sentence with:

Additionally, we analyze the co-follower, co-share, and co-retweet networks of 5000 Twitter users and observe an association between conservative ideology and misinformation exposure. Finally, we find that estimated ideological extremity is associated with more misinformation exposure to a greater extent for users estimated to be conservative than for users estimated to be liberal.

This has been corrected in both the PDF and HTML versions of the Article.

