# Supplementary material for: Measuring exposure to misinformation from political elites on Twitter
Source: Nat Commun. 2022 Nov 21;13:7144. doi: 10.1038/s41467-022-34769-6 (PMC9681735; doi:10.1038/s41467-022-34769-6)
Supplement: Supplementary file 1 — Supplementary Information [file 41467_2022_34769_MOESM1_ESM.pdf]

Supplementary Information

Measuring exposure to misinformation from political elites on Twitter

Mohsen Mosleh*

Management Department, University of Exeter Business School, United Kingdom; Alan Turing Institute, United Kingdom; Sloan School of Management, Massachusetts Institute of Technology, United States; m.mosleh@exeter.ac.uk

David G. Rand

Sloan School of Management, Massachusetts Institute of Technology, Institute for Data, Systems, and Society, Massachusetts Institute of Technology, Department of Brain and Cognitive Sciences, Massachusetts Institute of Technology, United States, drand@mit.edu

1 SUPPLEMENTARY FIGURES

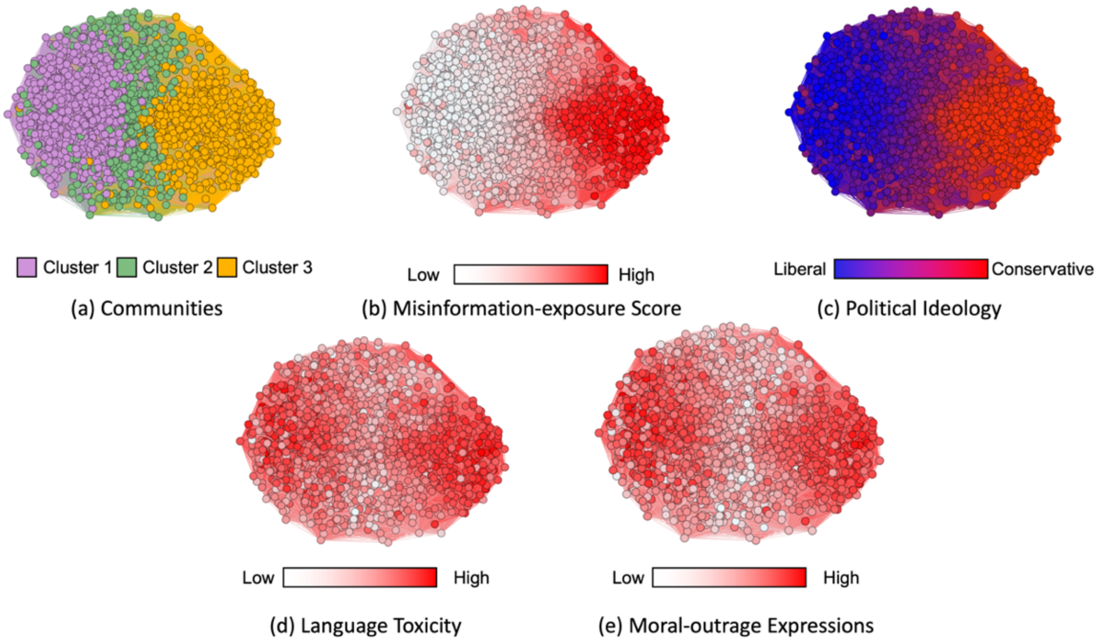

Supplementary Figure 1. Co-follower network*. Nodes represent Twitter accounts followed by at least 200 users in our dataset and edges are weighted based on common followers. (a) Separate colors represent different clusters using community detection algorithms [1]. (b) The intensity of color of each node shows the average misinformation-exposure score of its followers (darker = higher misinformation-exposure score). (c) Nodes' color represents the average estimated ideology of the followers (red: conservative, blue: liberal). (d) Average language toxicity used by the followers (darker = more toxic language use). (e) Average moral-outrage expressions (darker=more expressions of moral-outrage). Nodes are positioned using directed-force layout based on the weighted network. See Supplementary Table 7 for average characteristics of users who followed accounts in each cluster and Supplementary Table 8 for top 10 accounts in each cluster.*

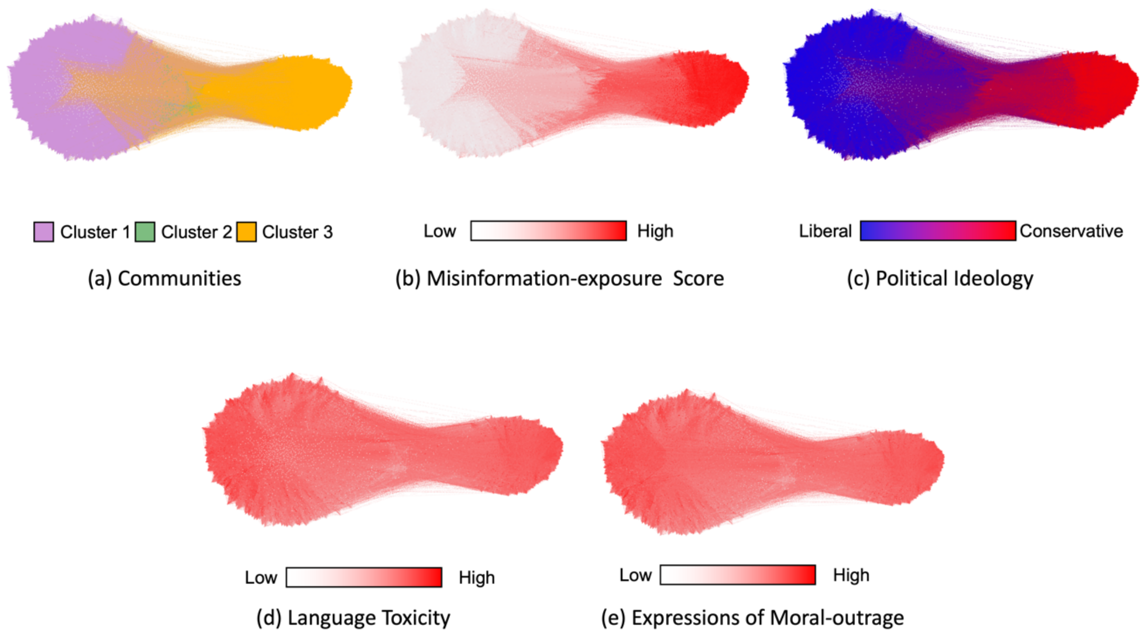

Supplementary Figure 2. Co-retweet network. *Nodes represent Twitter accounts retweeted by at least 20 users in our dataset and edges are weighted based on the number of common users who retweeted those accounts. (a) Separate colors represent different clusters using community detection algorithms [1]. (b) The intensity of color of each node shows the average misinformation-exposure score of the users who retweeted the accounts (darker = higher misinformation-exposure score). (c) Nodes' color represents the average estimated ideology of the users who retweeted the accounts (red: conservative, blue: liberal). (d) Average language toxicity used by the users who retweeted the accounts (darker = more toxic language use). (e) Average moral-outrage expressions (darker=more expressions of moral-outrage). Nodes are positioned using directed-force layout based on the weighted network. See Supplementary Table 9 for average characteristics of users who retweeted accounts in each cluster and Supplementary Table 10 for top 10 accounts in each cluster.*

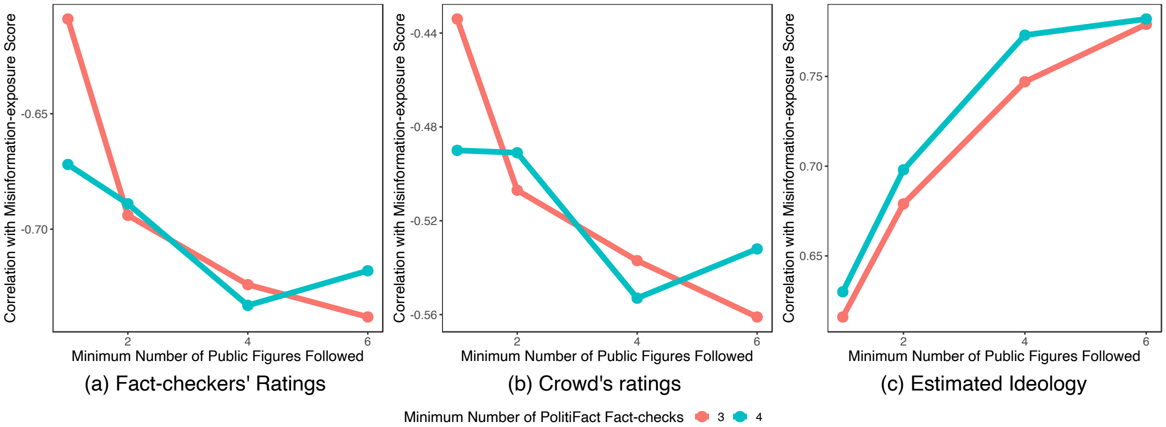

Supplementary Figure 3*. Robustness of results for various minimum number of elite accounts followed and minimum number of fact-checks by PolitiFact. We created separate lists of associated Twitter accounts for public figures with a minimum of 3 versus 4 PolitiFact fact-checks. Next, for each list of accounts, we generated separate random samples of 5,000 Twitter users who follow a minimum number of 1, 2, 4, or 6 falsity-rated accounts. Shown here are Pearson's correlation coefficients between misinformation-exposure score and (a) quality of content shared as measured by professional fact-checkers trustworthiness, (b) quality of content shared as measured by crowd trustworthiness, and (c) estimated political ideology. All correlations are significant (p<0.001 two-tailed). Across all three panels, while increasing the number of fact-checkers required for public figures or organizations to be included had little effect, increasing the minimum number of public figures followed (and thus increasing the precision of the misinformation-exposure score estimates) yields stronger correlations. Thus, when using this tool, one must make a trade-off between using a lower threshold for the number of followed accounts and being able to determine scores for a larger fraction of users (see Supplementary Figure 4) or using a higher threshold and achieving less noisy estimates for each user. Source data are provided as a Source Data file.*

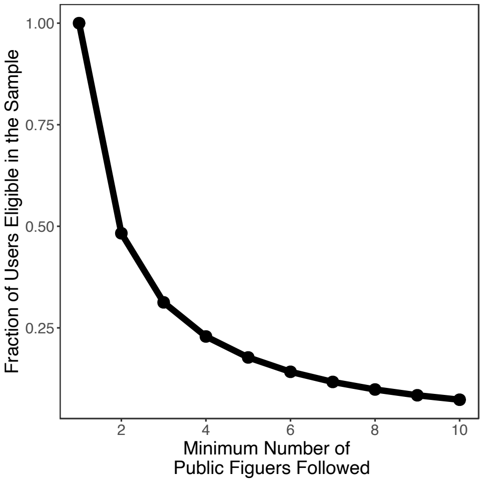

Supplementary Figure 4. Fraction of users within our sample (N= 122,562,681) for whom we can calculate a Misinformation-exposure Score. *Shown here is the fraction of our sample depending on what we set as the minimum number of rated accounts they must follow to be included. Source data are provided as a Source Data file.*

2 SUPPLEMENTARY TABLES

Supplementary Table 1. *Regression models predicting misinformation-exposure score using fact-checkers ratings and crowd's ratings of content users shares and their estimated political ideology. All statistical tests are two-tailed (p<.1, *p<0.05, **p<0.01, ***p<0.001)*

	**Dependent variable**	**Independent variable**	
**Model 1**	Fact-checkers ratings	Intercept	-0.037** (0.012)
		Misinformation-exposure score	-0.728*** (0.013)
**Model 2**	Crowd ratings	Intercept	-0.027· (0.015)
		Misinformation-exposure score	-0.540*** (0.015)
**Model 3**	Fact-checkers ratings	Intercept	-0.039** (0.013)
		Misinformation-exposure score	-0.712*** (0.020)
		Estimated political ideology	-0.021 (0.019)
**Model 4**	Crowd ratings	Intercept	-0.026· (0.015)
		Misinformation-exposure score	-0.565*** (0.024)
		Estimated political ideology	0.030 (0.023)
**Model 5**	Estimated political ideology	Intercept	-0.001 (0.010)
		Misinformation-exposure score	0.747*** (0.010)

Supplementary Table 2. *Regression models predicting language toxicity and express of moral-outrage using misinformation- exposure score and users' estimated political ideology. All statistical tests are two-tailed (·p<.1, *p<0.05, **p<0.01, ***p<0.001)*

	**Dependent variable**	**Independent variable**	
**Model 1**	Language toxicity	Intercept	0.002<br>(0.015)
		Misinformation-exposure score	0.129***<br>(0.015)
**Model 2**	Expression of moral outrage	Intercept	0.002<br>(0.015)
		Misinformation-exposure score	0.107***<br>(0.023)
**Model 3**	Language toxicity	Intercept	0.002<br>(0.015)
		Misinformation-exposure score	0.319***<br>(0.023)
		Estimated political ideology	-0.246***<br>(0.023)
**Model 4**	Expression of moral outrage	Intercept	0.003<br>(0.015)
		Misinformation-exposure score	0.329***<br>(0.023)
		Political ideology	-0.290***<br>(0.023)

Supplementary Table 3. *Robustness of results using estimating political ideology based on media sharing [2]. Regression models predicting misinformation-exposure score using fact-checkers ratings and crowd's ratings of content users shares and their estimated political ideology using media sharing. All statistical tests are two-tailed. (p<.1, *p<0.05, **p<0.01, ***p<0.001)*

	**Dependent variable**	**Independent variable**	**Estimating political ideology using accounts followed**	**Estimating political ideology using media sharing**
Model 1	Fact-checkers ratings	Intercept	-0.037** (0.012)	-0.037** (0.012)
		Misinformation-exposure score	-0.728*** (0.013)	-0.728*** (0.013)
Model 2	Crowd ratings	Intercept	-0.027· (0.015)	-0.027· (0.015)
		Misinformation-exposure score	-0.540*** (0.015)	-0.540*** (0.015)
Model 3	Fact-checkers ratings	Intercept	-0.039** (0.013)	-0.02· (0.012)
		Misinformation-exposure score	-0.712*** (0.02)	-0.457*** (0.017)
		Political ideology	-0.021 (0.019)	-0.373*** (0.017)
Model 4	Crowd ratings	Intercept	-0.026· (0.015)	-0.014 (0.015)
		Misinformation-exposure score	-0.565*** (0.024)	-0.366*** (0.022)
		Political ideology	0.03 (0.023)	-0.242*** (0.022)
Model 5	Political ideology	Intercept	-0.001 (0.010)	-0.05*** (0.013)
		Misinformation-exposure score	0.746*** (0.010)	0.695*** (0.013)

Supplementary Table 4. *Robustness of results for various functional forms mapping fact-checking categories to veracity scores. Mapping function 1 used in the main text: (True, Mostly True, Half True, Mostly False, False, Pants on Fire) = (5/5, 4/5, 3/5, 2/5, 1/5, 0), Mapping function 2 (True, Mostly True, Half True, Mostly False, False, Pants on Fire) =(1, 1, 0.5, 0, 0, 0); Mapping function 3 (True, Mostly True, Half True, Mostly False, False, Pants on Fire) =(1, 1, 0, 0, 0, 0); Mapping function 4 similar to the main text but not weighted by number of tweets of the public figure : (True, Mostly True, Half True, Mostly False, False, Pants on Fire) = (5/5, 4/5, 3/5, 2/5, 1/5, 0); Results shown are the coefficient and p-value of linear regression models predicting misinformation-exposure scores. All statistical tests are two-tailed.*

*All mapping functions are weighted by the number tweets made by the elites except mapping function 4. (·p<.1, *p<0.05, **p<0.01, ***p<0.001)*

	Dependent variable	Independent variable	Mapping function1	Mapping function 2	Mapping function 3	Mapping function 4
**Model 1**	Fact-checkers ratings	Intercept	-0.037**<br>(0.012)	-0.04**<br>(0.013)	-0.038**<br>(0.013)	-0.042**<br>(0.012)
		Misinformation-exposure score	-0.728***<br>(0.013)	-0.714***<br>(0.013)	-0.726***<br>(0.013)	-0.73***<br>(0.012)
**Model 2**	Crowd ratings	Intercept	-0.027·<br>(0.015)	-0.03·<br>(0.015)	-0.028·<br>(0.015)	-0.031·<br>(0.015)
		Misinformation-exposure score	-0.54***<br>(0.015)	-0.527***<br>(0.016)	-0.537***<br>(0.015)	-0.543***<br>(0.015)
**Model 3**	Fact-checkers ratings	Intercept	-0.039**<br>(0.013)	-0.046***<br>(0.013)	-0.04**<br>(0.013)	-0.041**<br>(0.012)
		Misinformation-exposure score	-0.712***<br>(0.02)	-0.647***<br>(0.019)	-0.714***<br>(0.02)	-0.76***<br>(0.02)
		Political ideology	-0.021<br>(0.019)	-0.086***<br>(0.019)	-0.016<br>(0.019)	0.036·<br>(0.02)
**Model 4**	Crowd ratings	Intercept	-0.026·<br>(0.015)	-0.032·<br>(0.015)	-0.027.<br>(0.015)	-0.028·<br>(0.015)
		Misinformation-exposure score	-0.565***<br>(0.024)	-0.508***<br>(0.024)	-0.565***<br>(0.025)	-0.61***<br>(0.025)
		Political ideology	0.03<br>(0.023)	-0.025<br>(0.022)	0.033<br>(0.023)	0.08**<br>(0.024)
**Model 5**	Political ideology	Intercept	-0.001<br>(0.010)	0.001<br>(0.010)	0.001<br>(0.010)	0.001<br>(0.010)
		Misinformation-exposure score	0.746***<br>(0.010)	0.728***<br>(0.010)	0.755***<br>(0.010)	0.772***<br>(0.010)

Supplementary Table 5. *Robustness of results when including only Twitter accounts related to public political figures (excluding accounts related to organizations) and when excluding elites with multiple Twitter accounts. To identify news organizations, we used type of the elite from the PolitiFact data and only included elites classified as "person". Results shown are the coefficient and p-value of linear regression models predicting misinformation-exposure scores. All statistical tests are two-tailed. (·p<.1, *p<0.05, **p<0.01, ***p<0.001).*

	**Dependent variable**	**Independent variable**	**Not Excluding anyone**	**Excluding news organizations' Twitter accounts**	**Excluding elites with multiple Twitter accounts**
**Model 1**	Fact-checkers ratings	Intercept	-0.037** (0.012)	-0.038** (0.013)	-0.036** (0.013)
		Misinformation-exposure score	-0.728*** (0.013)	-0.71*** (0.013)	-0.721*** (0.013)
**Model 2**	Crowd ratings	Intercept	-0.027· (0.015)	-0.028· (0.015)	-0.027· (0.015)
		Misinformation-exposure score	-0.54*** (0.015)	-0.519*** (0.015)	-0.533*** (0.016)
**Model 3**	Fact-checkers ratings	Intercept	-0.039** (0.013)	-0.04** (0.013)	-0.04** (0.013)
		Misinformation-exposure score	-0.712*** (0.02)	-0.689*** (0.021)	-0.684*** (0.02)
		Political ideology	-0.021 (0.019)	-0.026 (0.02)	-0.049. (0.019)
**Model 4**	Crowd ratings	Intercept	-0.026· (0.015)	-0.028· (0.016)	-0.027· (0.015)
		Misinformation-exposure score	-0.565*** (0.024)	-0.531*** (0.025)	-0.539*** (0.024)
		Political ideology	0.03 (0.023)	0.014 (0.024)	0.006 (0.023)
**Model 5**	Political ideology	Intercept	-0.001 (0.010)	-0.001 (0.01)	0 (0.010)
		Misinformation-exposure score	0.746*** (0.010)	0.759*** (0.010)	0.737*** (0.010)

Supplementary Table 6. *Correlation of quality of content shared and misinformation exposures score with users' characteristics. Results shown are the coefficient of Pearson correlation. All statistical tests are two-tailed. All correlations are significant (p<0.001 Pearson correlation). The magnitude of correlations for misinformation exposure score are significantly different from those for fact-checkers ratings and crowd ratings (p<0.001 using confidence intervals to compare correlations; [3]).*

	**Estimated political ideology**	**Use of toxic language**	**Expressions of moral outrage**
**Misinformation exposure score**	0.75	0.13	0.11
**Misinformation sharing (fact-checker ratings)**	-0.57	-0.25	-0.25
**Misinformation sharing (crowd ratings)**	-0.40	-0.19	-0.20

Supplementary Table 7. *Average characteristics of users who followed accounts in each cluster in the co-follower network. Misinformation-exposure score, use of toxic language, expression of moral-outrage differed significantly across clusters, even when controlling for average ideology score (p<0.001 with and without ideology control, Tukey Honest Significant Differences test; all statistical tests are two-tailed).*

	Number of nodes in cluster	Estimated political ideology	Misinformation exposure score	Use of toxic language	Expressions of moral-outrage
**Cluster 1**	898	-0.475	0.3767	0.1953	0.2162
**Cluster 2**	404	0.279	0.4104	0.1782	0.1779
**Cluster 3**	555	1.4266	0.5102	0.1997	0.2161

Supplementary Table 8. Top accounts in each cluster within the co-follower network*. For each cluster the table shows the 10 accounts in each cluster with the largest number of followers amongst our sample in descending order. Source data are provided as a Source Data file.*

Cluster 1	Cluster 2	Cluster 3
Barack Obama	The New York Times	The White House 45 Archived
Joe Biden	The Associated Press	President Trump 45 Archived
Hillary Clinton	The Washington Post	Melania Trump 45 Archived
Kamala Harris	CNN	Vice President Mike Pence Archived
Nancy Pelosi	CNN Breaking News	Kayleigh McEnany 45 Archived
Elizabeth Warren	The Wall Street Journal	Fox News
Rachel Maddow MSNBC	Bill Clinton	Ted Cruz
Bernie Sanders	BBC Breaking News	Sean Hannity
Elizabeth Warren	BBC News (World)	Ivanka Trump

Supplementary Table 9. *Average characteristics of users who retweeted accounts in each cluster in the co-retweet network. Misinformation-exposure score, use of toxic language, expression of moral-outrage differed significantly across clusters, even when controlling for average ideology score (p<0.001 with and without ideology control, Tukey Honest Significant Differences test; all statistical tests are two-tailed)*

	**Number of nodes in cluster**	**Estimated political ideology**	**Misinformation exposure score**	**Use of toxic language**	**Expressions of moral-outrage**
**Cluster 1**	3,996	-0.829	0.370	0.204	0.239
**Cluster 2**	159	0.348	0.422	0.169	0.175
**Cluster 3**	2,035	1.54	0.534	0.210	0.243

Supplementary Table 10. *Top accounts in each cluster within the co-retweet network. For each cluster the table shows the 10 accounts in each cluster with the largest number of followers amongst our sample in descending order.*

**Cluster 1**	**Cluster 2**	**Cluster 3**
The New York Times	The Hill	The White House
CNN	SportsCenter	Charlie Kirk
The Washington Post	ESPN	Fox News
Barack Obama	Bleacher Report	Donald Trump Jr.
ABC News	Mashable	James Woods,
Kyle Griffin	MLB	ULTRA POSO
Joe Biden	AFP News Agency	Ted Cruz
NBC News	Darren Rovell	The Wall Street Journal
The Associated Press	Adam Schefter	Ryan Fournier

Supplementary Table 11. *Regression model predicting misinformation-exposure score using estimated extremity of political ideology, political ideology (0: Liberal, 1: Conservative), and their interaction.*

	**Estimated Ideology using accounts followed**	**Estimated Ideology using media shared**
**Intercept**	-0.824***<br>(0.012)	-0.640***<br>(0.016)
**Estimated Extremity**	0.050**<br>(0.012)	0.070***<br>(0.018)
**Ideology (1:Conservative)**	1.22***<br>(0.015)	1.175***<br>(0.024)
**Estimated Extremity X Estimated Ideology**	0.756***<br>(0.015)	0.415***<br>(0.024)

Supplementary Table 12 . *Regression model predicting (a) language toxicity and (b) expressions of moral outrage using estimated political ideology extremity, political ideology (0: Liberal, 1: Conservative), and their interaction.*

	**Language toxicity**	**Expressions of moral outrage**
**Intercept**	0.099**<br>(0.026)	0.143***<br>(0.025)
**Estimated Extremity**	0.109**<br>(0.025)	0.131***<br>(0.025)
**Estimated Ideology: Conservative**	-0.150***<br>(0.032)	-0.216***<br>(0.032)
**Estimated Extremity X Estimated Ideology**	0.100**<br>(0.032)	0.105***<br>(0.031)

SUPPLEMENTARY REFERENCES

[1] Blondel, V. D., Guillaume, J.-L., Lambiotte, R. and Lefebvre, E. Fast unfolding of communities in large networks. *Journal of statistical mechanics: theory and experiment*, 2008, 10 (2008), P10008.
[2] Eady, G., Nagler, J., Guess, A., Zilinsky, J. and Tucker, J. A. How many people live in political bubbles on social media? Evidence from linked survey and Twitter data. *Sage Open*, 9, 1 (2019), 2158244019832705.
[3] Zou, G. Y. Toward using confidence intervals to compare correlations. *Psychological methods*, 12, 4 (2007), 399.
