# Supplementary material for: Measuring exposure to misinformation from political elites on Twitter
Source: Nat Commun. 2022 Nov 21;13:7144. doi: 10.1038/s41467-022-34769-6 (PMC9681735; doi:10.1038/s41467-022-34769-6)
Supplement: Supplementary file 3 — Reporting Summary [file 41467_2022_34769_MOESM3_ESM.pdf]

nature portfolio

Corresponding author(s): Mohsen Mosleh

Last updated by author(s): Oct 6, 2022

Reporting Summary

Nature Portfolio wishes to improve the reproducibility of the work that we publish. This form provides structure for consistency and transparency in reporting. For further information on Nature Portfolio policies, see our Editorial Policies and the Editorial Policy Checklist.

Statistics

For all statistical analyses, confirm that the following items are present in the figure legend, table legend, main text, or Methods section.

n/a	Confirmed	
☐	☒	The exact sample size (*n*) for each experimental group/condition, given as a discrete number and unit of measurement
☒	☐	A statement on whether measurements were taken from distinct samples or whether the same sample was measured repeatedly
☐	☒	The statistical test(s) used AND whether they are one- or two-sided<br>*Only common tests should be described solely by name; describe more complex techniques in the Methods section.*
☐	☒	A description of all covariates tested
☒	☐	A description of any assumptions or corrections, such as tests of normality and adjustment for multiple comparisons
☐	☒	A full description of the statistical parameters including central tendency (e.g. means) or other basic estimates (e.g. regression coefficient) AND variation (e.g. standard deviation) or associated estimates of uncertainty (e.g. confidence intervals)
☐	☒	For null hypothesis testing, the test statistic (e.g. *F*, *t*, *r*) with confidence intervals, effect sizes, degrees of freedom and *P* value noted<br>*Give P values as exact values whenever suitable.*
☒	☐	For Bayesian analysis, information on the choice of priors and Markov chain Monte Carlo settings
☒	☐	For hierarchical and complex designs, identification of the appropriate level for tests and full reporting of outcomes
☒	☐	Estimates of effect sizes (e.g. Cohen's *d*, Pearson's *r*), indicating how they were calculated

Our web collection on statistics for biologists contains articles on many of the points above.

Software and code

Policy information about availability of computer code

Data collection	All data were collected using Python 3.8 and Tweepy 4.8.0; community detection was done in Python "community" library 0.15
Data analysis	All analyses where don in Python 3.8 and R 4.0.4. All code used to generate the results are available on https://osf.io/5283b/

For manuscripts utilizing custom algorithms or software that are central to the research but not yet described in published literature, software must be made available to editors and reviewers. We strongly encourage code deposition in a community repository (e.g. GitHub). See the Nature Portfolio guidelines for submitting code & software for further information.

Data

Policy information about availability of data

All manuscripts must include a data availability statement. This statement should provide the following information, where applicable:

- Accession codes, unique identifiers, or web links for publicly available datasets
- A description of any restrictions on data availability
- For clinical datasets or third party data, please ensure that the statement adheres to our policy

Twitter data contains identifiable information -- and for confidentiality reasons -- are only available upon request. The Twitter data are available under restricted access for research purposes, access can be obtained by writing to the authors.

Human research participants

Policy information about studies involving human research participants and Sex and Gender in Research.

Reporting on sex and gender	We did not collected sex and gender of the users.
Population characteristics	See above
Recruitment	This was an observational study where we used the Twitter API to create a sample of 5000 users who followed political figures and organizations fact-checked by PolitiFact. We used the Twitter API to identify those accounts based on accounts they followed.
Ethics oversight	Our study received a waiver from an ethics review by the MIT Committee on the Use of Humans as Experimental Subjects (COUHES) protocol E-3973.

Note that full information on the approval of the study protocol must also be provided in the manuscript.

Field-specific reporting

Please select the one below that is the best fit for your research. If you are not sure, read the appropriate sections before making your selection.

☐ Life sciences ☒ Behavioural & social sciences ☐ Ecological, evolutionary & environmental sciences

For a reference copy of the document with all sections, see nature.com/documents/nr-reporting-summary-flat.pdf

Behavioural & social sciences study design

All studies must disclose on these points even when the disclosure is negative.

Study description	From a database of professional fact-checks by PolitiFact, falsity scores can be calculated for 816 public figures. We then assign users an elite misinformation-exposure score by averaging the falsity scores of the public figures they follow. We show that users' misinformation-exposure scores are negatively correlated with the quality of news they share (based on ratings from both professional fact-checkers and a politically-balanced crowd of laypeople), and positively correlated with conservative ideology. Additionally, we analyze the co-follower and the co-share network of 5,000 Twitter users and find an ideological asymmetry
Research sample	We created a list of followers of all accounts for whom PolitiFact provided a falsity score and we then randomly sampled from those followers. Our sample is not representative of Twitter users yet focus on those who followed political figures and organizations whose statements were fact-checked by political thus we could calculate the misinformation-exposure score for them.
Sampling strategy	We randomly sampled 5,000 Twitter users who followed at list 3 public figures for whom we had falsity scores from Politi Fact. We provided further robustness for this choice. We chose N=5,000 for computational tractability reasons.
Data collection	We combined publicly available fact-checking data from Politi Fact for public figures. We also used Twitter Academic API to collect public data from Twitter for followers of those public figures. The study was exploratory and the researchers were not blind to the hypothesis.
Timing	Politi Fact data ratings was collected on Oct 28 2020. Twitter data was collected on July 23 2021
Data exclusions	We could not collect data for 837 out of 5000 Twitter accounts since they were protected accounts, did have any tweet, or did not exist anymore.
Non-participation	This was observational study thus the users were not provided an option not to participate.
Randomization	This was an observational study thus we did not do any randomization across conditions.

Reporting for specific materials, systems and methods

We require information from authors about some types of materials, experimental systems and methods used in many studies. Here, indicate whether each material, system or method listed is relevant to your study. If you are not sure if a list item applies to your research, read the appropriate section before selecting a response.

Materials & experimental systems

n/a	Involved in the study
☒	☐ Antibodies
☒	☐ Eukaryotic cell lines
☒	☐ Palaeontology and archaeology
☒	☐ Animals and other organisms
☒	☐ Clinical data
☒	☐ Dual use research of concern

Methods

n/a	Involved in the study
☒	☐ ChIP-seq
☒	☐ Flow cytometry
☒	☐ MRI-based neuroimaging
